# Assessing Spillover Effects of Medications for Opioid Use Disorder on HIV Risk Behaviors among a Network of People Who Inject Drugs

**DOI:** 10.3390/stats7020034

**Published:** 2024-06-19

**Authors:** Joseph Puleo, Ashley Buchanan, Natallia Katenka, M. Elizabeth Halloran, Samuel R. Friedman, Georgios Nikolopoulos

**Affiliations:** 1 Department of Computer Science & Statistics, University of Rhode Island, Kingston, RI 02881, USA; 2 Department of Pharmacy Practice & Clinical Research, University of Rhode Island, Kingston, RI 02881, USA; 3 Vaccine and Infectious Disease Division, Fred Hutchinson Cancer Center, Seattle, WA 98109, USA; 4 Department of Biostatistics, University of Washington, Seattle, WA 98195, USA; 5 Department of Population Health, NYU Grossman School of Medicine, New York, NY 10016, USA; 6 Medical School, University of Cyprus, 1678 Nicosia, Cyprus

**Keywords:** causal inference, networks, spillover, community detection, Human Immunodeficiency Virus, People Who Inject Drugs

## Abstract

People who inject drugs (PWID) have an increased risk of HIV infection partly due to injection behaviors often related to opioid use. Medications for opioid use disorder (MOUD) have been shown to reduce HIV infection risk, possibly by reducing injection risk behaviors. MOUD may benefit individuals who do not receive it themselves but are connected through social, sexual, or drug use networks with individuals who are treated. This is known as spillover. Valid estimation of spillover in network studies requires considering the network’s community structure. Communities are groups of densely connected individuals with sparse connections to other groups. We analyzed a network of 277 PWID and their contacts from the Transmission Reduction Intervention Project. We assessed the effect of MOUD on reductions in injection risk behaviors and the possible benefit for network contacts of participants treated with MOUD. We identified communities using modularity-based methods and employed inverse probability weighting with community-level propensity scores to adjust for measured confounding. We found that MOUD may have beneficial spillover effects on reducing injection risk behaviors. The magnitudes of estimated effects were sensitive to the community detection method. Careful consideration should be paid to the significance of community structure in network studies evaluating spillover.

## 1. Introduction

People who inject drugs (PWID) are a population with a high risk for HIV infection via sexual and injection risk behaviors. PWID often have limited access to harm reduction resources, and engagement in harm reduction is further compounded by stigma associated with HIV and drug use, incarceration, unstable housing, and socioeconomic factors [1–4]. Currently, there are three medications approved by the Food and Drug Administration for the treatment of opioid use disorder (buprenorphine, methadone, and naltrexone) [5]. Unfortunately, large treatment gaps remain for opioid use disorder (OUD), and less than 20% of people with OUD received medication for opioid use disorder (MOUD) in the US in 2022 [6,7]. Individuals treated for OUD are less likely to engage in injection risk behavior [8–13]. Furthermore, MOUD may benefit individuals beyond those treated and impact other individuals in close physical or social proximity who may not be able or willing to receive the treatment themselves [14–17]. Few studies have assessed the effect of MOUD on reducing HIV injection risk behavior among PWID and their risk contacts in a network study [18]. This is broadly known as spillover, meaning that the treatment (intervention or exposure) of one individual could possibly affect the outcome of another individual. Importantly, PWID are often part of social, drug use, or sexual networks, where individuals are connected by social interaction, sexual, non-injection drug use, or injection drug use behaviors [19]. Patients receiving MOUD may share these medications with their network contacts who experience withdrawal and related symptoms, and subsequently also reduce those individuals’ drug use and injection risk behaviors, such as sharing syringes [20–24]. MOUD recipients may also receive information on harm reduction, which, in turn, can also benefit their contacts if this information is disseminated. Due to such possible spillover, increasing MOUD uptake in a PWID network may have a protective effect against HIV injection risk behaviors beyond treated individuals. MOUD is known to reduce injection risk behaviors among those who are treated and may also protect treated individuals’ network contacts and possibly their contacts’ contacts from HIV injection risk behaviors. This may protect against HIV acquisition among individuals who are HIV seronegative or onward HIV transmission if they are HIV-positive. By estimating spillover, we can gain a more complete understanding of its potential benefits in reducing HIV injection risk behaviors among not only PWID who are treated with MOUD themselves but also their contacts.

The causal inference methods used in this study to assess spillover rely on a partial interference assumption [25,26], where non-overlapping communities or clusters can be identified in the study data, and spillover is assumed to possibly occur between individuals within communities (or “interference sets”) but not between individuals in different communities. We define communities as groups of individuals who are connected through sexual partnerships, drug use partnerships, or frequenting the same venues for drug use, with only sparse connections to individuals in other groups.

In the setting of HIV risk networks, these separate and independent groups are often challenging to identify, and methods for assessing spillover in networks where interference sets are not known a priori are not well established in the causal inference literature [25–34]. Other studies of spillover defined interference sets based on an agglomerative spatial clustering method when group membership was not predefined [32–34]. However, these studies did not consider the impact of different community detection methods on the analysis results. Therefore, new approaches are needed to evaluate whether communities identified using community detection methods represent a meaningful structure relevant for spillover in network studies among PWID. In this paper, we consider the significance of the community structure to be the degree to which a true underlying community structure actually exists in the network, where communities are distinct from one another with many connections within them and few between them. In a network with a non-significant community structure, the network can be thought of as one large community without truly separate or distinct groupings within it.

This study focuses on the Transmission Reduction Intervention Project (TRIP), which was a sociometric study of PWID and their contacts conducted in Athens, Greece, from 2013 to 2015 [35]. We first identified communities in the PWID network using a modularity-based community detection method [27,28]. We then employed an inverse probability weighted approach with community-level propensity scores to adjust for measured time-invariant confounding. To account for uncertainty in the community structures ascertained using two community detection methods, the cluster-fast-greedy method and the leading-eigenvector method, we conducted a sensitivity analysis to compare the causal effect estimates for each community detection method. In this work, we highlight the importance of analyzing the community structure when conducting causal analysis in the presence of spillover in network studies.

The rest of the paper is organized as follows. In Section 2, we provide details about TRIP (Section 2.1), a brief overview of causal inference methodology in the presence of spillover and the community detection methods (Section 2.2), the notation for the statistical methods (Section 2.3), and a detailed explanation of the parameters of interest and their corresponding estimators (Section 2.4). We explain our assessment of the significance of the community structure (Section 2.5), and the application of this approach to the TRIP data (Section 2.6). Section 3 provides the results of the evaluation of spillover and the analysis of the community structure. In Section 4 we discuss our findings, conclusions, and directions for further research.

## 2. Materials and Methods

We assessed the spillover effects of MOUD receipt or prescription (referred to as treatment hereafter) on subsequent HIV injection risk behavior using a causal inference approach [25,29]. We also employed concepts from information theory [27,28,36] to assess the significance of the community structure in the network to possibly explain any sensitivity of causal effect estimates to the community detection method.

### 2.1. Motivating Study

The primary aim of TRIP was to develop and implement novel approaches to recruit people who had recently become HIV positive and refer them to care while they were likely in the acute/early stage of infection (i.e., highly infectious stage) [19,35,37]. The study then recruited the participants’ contacts and possibly the contacts’ contacts. In the event that there was a person in close proximity in the observed network who recently became HIV positive, the study also aimed to alert their network members to take extra precautions to avoid or reduce HIV risk behavior for six months [19,35,37].

Interviews were conducted with participants to collect data, including demographics, sexual and injection risk behaviors, and substance use treatment, including behavioral therapy and MOUD in the prior 6 months. Follow-up interviews were conducted approximately 6 months after each participant’s baseline interview. Initial participants, known as “seeds,” were HIV positive and referred to TRIP by collaborating HIV testing and treatment sites. The recruitment of the seeds’ contacts was conducted using two-wave contact tracing. Network connections between participants (also referred to as “edges”) were based on their contact in the 6 months prior to baseline. Two participants shared an edge if they injected drugs together or had sex; injected or had sex in each other’s presence; injected, used drugs or had sex with the same people; or were reported to have frequented the same venues to use drugs, have sex, or meet new sex partners. Their contacts who were enrolled in the study were then tested for HIV. After the initial two waves of contact tracing were completed for the seeds, two-wave contact tracing was conducted for those newly recruited who recently became HIV positive. Seventy-nine HIV-negative participants were enrolled in the study as a control group, and their network contacts were not traced, although they were connected to TRIP participants who were identified as contacts [19,35,37].

### 2.2. Causal Inference Methods and Assumptions

Our approach uses a potential outcomes framework. Average causal effects are identifiable in observational studies provided that the following three identifiability conditions are satisfied: treatment variation irrelevance, positivity, and exchangeability [38,39]. In general, treatment variation irrelevance means that the effect of the binary treatment on the binary outcome (HIV injection risk behavior) is unaffected by the type, dosing, or other variations of treatment (e.g., MOUD received is methadone versus buprenorphine); that is, there are only two versions of treatment (treated versus not treated) that map onto the two potential outcomes. Positivity means that the probability of MOUD treatment (and no treatment) is positive within levels of all combinations of the observed confounders. Exchangeability implies that treated and untreated participants are comparable, on average, within levels of measured confounders. In this context of an observed network with a non-randomized treatment, these assumptions extend to the community level. Community-level positivity means that within all levels of community-level confounders, there is a non-zero probability of all possible sets of MOUD treatment assignments within each community. Community-level exchangeability implies that communities with different sets of observed treatment allocation strategies are comparable, on average, within levels of measured community-level confounders.

In this study, we relaxed the assumption of no interference (or spillover) and allowed for partial interference, which can occur between participants within communities (or “interference sets”) but not between participants in different communities. We defined a community as a group of participants that are densely connected in the network, with only sparser connections to other groups [27,28]. Under this definition, each participant is part of a community with few connections to participants in other communities. Therefore, the spillover effects of MOUD treatment on any participant’s HIV injection risk behavior is assumed to be based on the MOUD treatment of participants in that person’s respective community rather than the MOUD treatment of participants in other communities. We also assumed stratified interference, meaning that spillover depends on the proportion of people treated in a community rather than those treated in the community [30,31].

Due to our partial interference assumption, clusters or groupings of participants in the observed network needed to be identified, and these are referred to as communities in the network science literature [27,28]. In our approach, we first identified connected components in the network: groups of connected participants with no connections to other groups. We then identified communities by applying modularity-based community detection to the observed network components using two widely used community detection algorithms that maximize modularity [27,28]. We defined modularity as the difference between the proportion of edges (i.e., connections between participants) within communities and the expected proportion of edges within communities if edges were assigned to pairs of nodes (i.e., participants) completely at random [28]. We first used the cluster-fast-greedy algorithm [40], which maximizes modularity by assigning each node in the network graph as the sole member of a community of size 1. The next step was to join pairs of communities and choose the set of pairings that resulted in the largest increase in modularity. This process of joining pairs of communities based on the largest increase in modularity was repeated until the modularity no longer increased. We then used the leading-eigenvector modularity maximization algorithm [41]. This algorithm utilized results from the eigen-analysis of certain matrices, particularly the modularity matrix of the network graph, to split the network into two communities such that modularity is maximized. The two communities were then subdivided further using the same process, which continued until modularity no longer increased [28].

### 2.3. Notation

We introduce the notion of a counterfactual treatment allocation strategy to define the four causal effects of interest. In a counterfactual scenario, the strategy α represents the counterfactual scenario in which participants in the community are treated with probability α, and we refer to this parameter as the treatment coverage in the community. We assume a Bernoulli individual group counterfactual treatment allocation strategy, where treatment is assigned independently within levels of the covariates according to (and possibly contrary to fact) strategy α [26]. We standardize the observed treatment vectors to a study population in which treatment assignment follows a Bernoulli distribution with probability α. We are not assuming that observed treatments are independent Bernoulli random variables; however, this distribution of treatment is used to define the counterfactual estimands [25,26,32].

Assume there are N communities and each community i has $n_{i}$ participants for i=1,2,…,N, then $Y_{ij},\;A_{ij}$, and $X_{ij}$ represent the observed outcome, observed treatment status, and the vector of baseline covariates for the $j^{th}$ participant in community $i.\;A_{i}$ is a vector of treatment allocations, and $X_{i}$ is a matrix of baseline covariates for members within community i. Let 𝒜(n) be the set of all vectors of possible treatment allocations of length n. For example, under a binary treatment, A takes the value a∈{0,1}, where a=0 represents having *not* been assigned MOUD treatment and a=1 represents having been assigned MOUD treatment. We denote the potential outcome for participant j in community i as $Y_{ij}\left(a_{ij},a_{i,-j}\right)=Y_{ij}\left(a_{i}\right)$ if the community received treatment vector $a_{i}$. When indexing the potential outcomes, we consider the treatment status of participant j in community i (denoted $a_{ij}$), as well as the treatment vector that includes the treatments of everyone else in community i excluding participant j (denoted $a_{i,-j}$).

We assume a community-level generalization of conditional exchangeability, i.e., $\text{Pr}\left(A_{i}=a_{i}|X_{i},Y_{ij}\left(a_{i}\right)\right)=\text{Pr}\left(A_{i}=a_{i}|X_{i}\right)$. By treatment variation irrelevance, $Y_{ij}\left(A_{i}\right)=Y_{ij}$. We also assume conditional positivity at the community level, i.e., $P\left(A_{i}=a_{i}|X_{i}\right)>0,\forall \;a_{i}\in 𝒜\left(n_{i}\right)$. To define the potential outcomes, we assume a Bernoulli individual group treatment allocation strategy, denoted as α. In a counterfactual scenario, α is the probability of any participant being independently assigned the treatment. The probability of the treatment vector for community i under allocation strategy α can be written as

(1) $$\pi_{i}\left(A_{i};\alpha \right)=\prod_{j=1}^{n_{i}}\alpha^{A_{ij}}(1-\alpha {)}^{1-A_{ij}},$$

and the probability of the treatment vector for community i which excludes participant j can be written as

(2) $$\pi_{i}\left(A_{i,-j};\alpha \right)=\prod_{k=1,k\neq j}^{n_{i}}\alpha^{A_{ik}}(1-\alpha {)}^{1-A_{ik}}.$$

We also introduce additional notation specific to the sensitivity analyses for the robustness of the estimated effects of the two community detection approaches (described in Section 2.5). Let C be the community structure in the network, i.e., the set of communities ascertained by maximizing modularity. We then let C(γ) be the community structure for a “perturbed” network, where the perturbed network is created by randomly reassigning a small proportion γ of edges. We denote the *variation of information* (VOI) for C and C(γ) as V(C,C(γ)), where the variation of information is defined as the sum of information needed to describe C given C(γ) and the information needed to describe C(γ) given C, normalized to a 0 to 1 scale [36]. A more detailed explanation of VOI can be found in the Appendix A. We create thirty perturbed networks allowing for thirty values of V(C,C(γ)) to be calculated, i.e., $𝒱(\gamma )=\left\{V\left(C,C_{1}(\gamma )\right),\ldots ,V\left(C,C_{30}(\gamma )\right)\right\}.𝒱(\gamma )$ is calculated for values of γ ranging from 0 to 1. Summary statistics, including the mean, minimum, and maximum, are denoted as $\overset{‾}{V}(\gamma )$, $V_{\text{min}}(\gamma )$, and $V_{\text{max}}(\gamma )$, respectively.

### 2.4. Estimands and Estimators

By employing a community-level inverse probability-weighted approach to adjust for measured confounding, we estimate the *direct*, *spillover*, *total*, and *overall* effects [31,42,43]. The direct effect is the difference in average potential outcomes under MOUD versus no MOUD with the same counterfactual treatment allocation in the community. This can be interpreted as the additional benefit of treatment beyond being in a community with a certain coverage level of the treatment. The spillover effect is the difference in average potential outcomes of an untreated participant under two different levels of MOUD coverage. The total effect is the difference in the average potential outcomes for treated participants under MOUD coverage level α versus untreated participants under MOUD coverage level $\alpha^{\prime }$, where $\alpha >\alpha^{\prime }$. The overall effect contrasts the average study population mean potential outcome under one treatment coverage (α) to the average study population mean potential outcome under another treatment coverage $\left(\alpha^{\prime }\right)$. We employ mixed effects logit models with a random effect for the community to estimate community-level treatment propensity scores [25,32]. We then estimate the difference of the average outcomes weighted by the inverse of the community-level treatment propensity score, thus consistently estimating the causal effects of interest [25,32].

When participant j is assigned to the treatment a∈{0,1} with probability α, the participant average potential outcome is defined by

(3) $${\overset{‾}{Y}}_{ij}(a;\alpha )=\sum_{a_{i,-j}\in 𝒜\left(n_{i}-1\right)}Y_{ij}\left(a_{ij},a_{i,-j}\right)\pi_{i}\left(a_{i,-j};\alpha \right).$$

The participant average potential outcome results from taking a weighted average of all of the participant j’s potential outcomes across the distribution of all possible treatment vectors for the other participants in community i under allocation α. The population average potential outcome under treatment a is then defined as

(4) $$\overset{‾}{Y}\left(a;\alpha \right)=\frac{1}{N}\sum_{i=1}^{N}\left\{\frac{1}{n_{i}}\sum_{j=1}^{n_{i}}{\overset{‾}{Y}}_{ij}\left(a;\alpha \right)\right\},$$

where we average the participant average potential outcome across all participants in community i, and then across the N communities. Thus, the direct effect can be represented as

(5) $$\bar{DE}(\alpha )=\overset{‾}{Y}(a=1;\alpha )-\overset{‾}{Y}(a=0;\alpha ),$$

the spillover effect can be represented as

(6) $$\bar{SE}\left(\alpha ,\alpha^{\prime }\right)=\overset{‾}{Y}(a=0;\alpha )-\overset{‾}{Y}\left(a=0;\alpha^{\prime }\right),$$

and the total effect can be represented as

(7) $$\bar{TE}\left(\alpha ,\alpha^{\prime }\right)=\overset{‾}{Y}\left(a=1;\alpha \right)-\overset{‾}{Y}\left(a=0;\alpha^{\prime }\right),$$

where $\alpha >\alpha^{\prime }$. The participant *marginal* average potential outcome is defined by

(8) $${\overset{‾}{Y}}_{ij}\left(\alpha \right)=\sum_{a_{i}\in 𝒜\left(n_{i}\right)}Y_{ij}\left(a_{i}\right)\pi_{i}\left(a_{i};\alpha \right).$$

The participant marginal average potential outcome results from taking a weighted average of all participant j’s potential outcomes across the distribution of all possible treatment vectors in community i under allocation *alpha*. The population average marginal potential outcome is then defined as

(9) $$\overset{‾}{Y}(\alpha )=\frac{1}{N}\sum_{i=1}^{N}\left\{\frac{1}{n_{i}}\sum_{j=1}^{n_{i}}{\overset{‾}{Y}}_{ij}(\alpha )\right\},$$

where we average the participant marginal average potential outcome across participants in community i, and then across the N communities. Thus, the overall effect can be represented as

(10) $$\bar{OE}\left(\alpha ,\alpha^{\prime }\right)=\overset{‾}{Y}(\alpha )-\overset{‾}{Y}\left(\alpha^{\prime }\right),$$

where $\alpha >\alpha^{\prime }$ [43].

To define the weights for the IPW estimator, we use the inverse of the community-level propensity score, where the propensity score is the probability of the observed treatment vector for a community conditional on baseline covariates. When the community-level propensity scores are known and the identifiability assumptions hold, these IPW estimators are unbiased; however, if the propensity scores are not known but estimated, these IPW estimators are consistent [25,32]. Because our study data come from an observational study, the propensity scores are not known. We first estimate the propensity scores for each participant using a mixed effects logit model with a random effect to account for dependency within communities. We then estimate the community-level propensity scores by integrating the estimated probability of observed treatment vectors in the community over the distribution of the random effects [25]. The community-level propensity score is

(11) $$f_{A|X}\left(A_{i}|X_{i}\right)=\int \prod_{j=1}^{n_{i}}h_{A|X}{\left(A_{ij}|X_{ij},b_{i}\right)}^{A_{ij}}{\left\{1-h_{A|X}\left(A_{ij}|X_{ij},b_{i}\right)\right\}}^{1-A_{ij}}f_{b}\left(b_{i}|\sigma_{b}^{2}\right)db_{i},$$

where $h_{A|X}\left(A_{ij}|X_{ij},b_{i}\right)=\text{Pr}\left(A_{ij}=1|b_{i},X_{ij}\right)$ is the inverse-logit of the generalized linear mixed regression model which estimates the participant-level propensity score for participant j in community $i,X_{ij}$ denotes baseline confounders for participant j in community i, and $b_{i}$ is a random effect for community i, which follows a normal distribution $f_{b}\left(b_{i}|\sigma_{b}^{2}\right)$ with mean 0 and variance $\sigma_{b}^{2}$. The IPW community-level average potential outcome and the marginal average potential outcome are obtained by the following estimators, respectively [25,32]:

(12) $${\hat{Y}}_{i}^{\text{IPW}}\left(a;\alpha \right)=\frac{\sum_{j=1}^{n_{i}}\pi_{i}\left(A_{i,-j};\alpha \right)A_{ij}Y_{ij}}{n_{i}f_{A|X}\left(A_{i}|X_{i}\right)},$$

(13) $${\hat{Y}}_{i}^{\text{IPW}}(\alpha )=\frac{\sum_{j=1}^{n_{i}}\pi_{i}\left(A_{i};\alpha \right)Y_{ij}}{n_{i}f_{A|X}\left(A_{i}|X_{i}\right)}.$$

We obtain the estimated population average potential outcomes and the population average marginal potential outcomes by averaging across communities. Therefore,

(14) $${\hat{Y}}^{\text{IPW}}(a;\alpha )=\frac{1}{N}\sum_{i=1}^{N}{\hat{Y}}_{i}^{\text{IPW}}(a,\alpha ),$$

and

(15) $${\hat{Y}}^{\text{IPW}}\left(\alpha \right)=\frac{1}{N}\sum_{i=1}^{N}{\hat{Y}}_{i}^{\text{IPW}}\left(\alpha \right).$$

The IPW estimators for the four causal effects of interest are as follows:

(16) $$\hat{DE}\left(\alpha \right)={\hat{Y}}^{\text{IPW}}\left(a=1;\alpha \right)-{\hat{Y}}^{\text{IPW}}\left(a=0;\alpha \right),$$

(17) $$\hat{SE}\left(\alpha ,\alpha^{\prime }\right)={\hat{Y}}^{\text{IPW}}\left(a=0;\alpha \right)-{\hat{Y}}^{\text{IPW}}\left(a=0;\alpha^{\prime }\right),$$

(18) $$\hat{TE}\left(\alpha ,\alpha^{\prime }\right)={\hat{Y}}^{\text{IPW}}\left(a=1;\alpha \right)-{\hat{Y}}^{\text{IPW}}\left(a=0;\alpha^{\prime }\right),$$

(19) $$\hat{OE}\left(\alpha ,\alpha^{\prime }\right)={\hat{Y}}^{\text{IPW}}(\alpha )-{\hat{Y}}^{\text{IPW}}\left(\alpha^{\prime }\right),\;\text{where}\;\alpha >\alpha^{\prime }.$$

We used robust estimators of the standard error (i.e., accounted for estimation of the weights) for each effect estimator to obtain 95% Wald-type confidence intervals [32,44].

Community detection is performed in R Studio using the “igraph” package [45]. The IPW estimators for the four causal effects of interest are calculated in R Studio using the “inferference” package [44]. We estimate these causal effects separately using communities defined by the community structure found from the cluster-fast-greedy method (CFG) [40] and the leading-eigenvector (LE) method [41]. We test the assumption of normality of random effects in the mixed effects models using an approach of Tchetgen Tchetgen and Coull (2006) [46].

### 2.5. Significance of the Community Structure

In a sensitivity analysis, we assess the robustness of the estimated effects to the two community detection approaches. We employ a method for assessing the significance of the community structure based on concepts from *information theory*, specifically variation of information (VOI). Karrer et al. [36] argue that the defining property of significant community structure is its robustness against small perturbations (i.e., random reassignment of a small proportion of edges to different pairs of nodes) in the network. VOI provides a measure of the amount by which the community structure is changed as a result of perturbing the network [36]. First, we ascertain the community structure (i.e., sets of non-overlapping communities) using modularity-based community detection. We then perturb the network by randomly reassigning a small proportion of edges. The modularity-maximizing community structure of this new perturbed network is then ascertained using community detection. The VOI is then calculated for the two community structures, providing a measure of sensitivity of the community structure to relatively small changes to the network (i.e., a small proportion of edges being randomly reassigned). This process is repeated many times on the TRIP network for proportions of edge reassignment ranging from 0 to 1. For comparison, we also perform this process on a “null network”, a randomly generated graph with the same degree sequence as the TRIP network (i.e., both networks had the same of distribution of edges per person) but that has no community structure (i.e., no distinguishable sets of densely connected participants with only sparse connections to other groups). This comparison allows for the assessment of a potential lack of significance in the community structure in the TRIP network and possibly a possible explanation for differences in causal effect estimates under the two community detection methods.

### 2.6. Analysis Methods in TRIP Study

TRIP aimed to develop novel approaches to recruit people who had recently become HIV positive and refer them to care while they were still in the acute/early stage of infection, and thus at a heightened risk of transmission. After identifying and treating persons rapidly after infection and then linking them to continued care for HIV treatment, the study then recruited their contacts and possibly contacts’ contacts [19,35,37]. To represent the PWID network in TRIP, we use a mathematical graph representation. The network graph G=(V;E) is comprised of a set of nodes (or vertices), V, where each node represents a study participant; and a set of edges, E, a set of connected pairs of nodes. In the TRIP study, two participants (i.e., two nodes in the network graph) were connected (i.e., shared an edge) if they had social contact with possible HIV risk behavior within the 6 months prior to baseline, including sexual contact, shared drug use, or were reported to have frequented the same venues. In our analysis, the treatment is defined as self-reported receipt of MOUD as part of a program or a prescription of MOUD in the 6 months prior to the baseline interview in TRIP. The treatment is represented as the binary variable A, where A=1 if the participant self-reported receipt or prescription of MOUD in the 6 months prior to the baseline interview and A=0 if they reported otherwise. The outcome is a self-reported engagement in any HIV injection risk behavior between the baseline and 6-month follow-up interview. We consider the following HIV injection risk behaviors: (1) shared a syringe that someone else had previously used to inject, (2) gave someone a syringe to use that the interviewed participant already injected with, (3) shared a cooker, filter, or rinsed water that someone else had previously used to inject, (4) gave someone a cooker, filter or rinsed water that the interviewed participant had previously used to inject, and (5) backloaded (“piggy-backed”) to share injection drugs. The outcome in our study is a binary variable denoted as Y, where Y=1 if the participant reported engaging in any of the five risk behaviors in the 6 months prior to the follow-up visit, and Y=0 if the participant reported *not* engaging in any of the injection risk behaviors listed above in the 6 months prior to the follow-up visit. We define treatment as a receipt or prescription for MOUD but do not have information available in the study to consider adherence.

The covariates included in this analysis to adjust for measured confounding are nationality, HIV status, recruitment type, ability to access health care when needed, injection drug use, daily use of alcohol, use of crack, use of heroin, daily use of cocaine, use of methamphetamines, marital status, employment status, education level, homelessness, sex, age, and self-reported injection risk behavior ascertained at the baseline visit. These were chosen based on other published studies that assessed the effect or association of MOUD on HIV risk behaviors and were known or suspected risk factors for the outcome, as well as consultation with the study team [47–52].

To address the correlation between variables and model convergence issues, variable selection for the propensity score models is performed in the following steps. We fit a logit model for MOUD treatment based on all baseline characteristics shown in Table 1 and examine the variance inflation factors (VIFs). None of the VIFs are large, and thus no variables are excluded based on VIFs. We then examine the pairwise association between all categorical variables. All variables that are associated with others in the model (i.e., chi-square test p-value < 0.05) are removed. Additional variables are removed due to model convergence issues related to small cell counts. Variables included in the propensity score models are age, marital status, nationality, crack use, daily alcohol use, ability to obtain medical care when needed, and baseline injection risk behavior.

Missing data for covariates and the outcome are handled using two imputation methods. We use a random forest method to impute the missing data for the covariates rather than removing participants with missing data from the network [53]. This method performs well for data that are missing completely at random (MCAR) or missing at random (MAR) [53]. Missing data for the outcome are imputed with a random draw from a conditional binomial distribution, where the parameter for the binomial distribution is estimated using a mixed effects logit model with a random effect for the communities. This imputation method allows for data that are MCAR or MAR [54].

Multiple sensitivity analyses are performed to assess the sensitivity of causal estimates to the missing outcome imputation method, as well as sensitivity to extreme community-level inverse probability weights (IPWs). The sensitivity of causal estimates to the missing data imputation is assessed using a best/worst-case scenario approach. For the best-case scenario, we impute the missing outcome as though participants who were prescribed MOUD treatment at baseline did not engage in HIV risk behaviors during the 6-month follow-up period, and participants who were not prescribed MOUD treatment did engage in HIV risk behaviors during the 6-month follow-up. For the worst-case scenario, we impute the missing outcome as though participants who were prescribed MOUD treatment at baseline did engage in HIV risk behaviors during the 6-month follow-up period, and participants who were not prescribed MOUD treatment did not engage in HIV risk behaviors during the 6-month follow-up. This approach provides the largest and smallest possible effect sizes compatible with the observed data [55]. Results and discussion of this sensitivity analysis can be found in the Appendix B.1. In an additional sensitivity analysis, missing data for the outcome are imputed with a random draw from a conditional binomial distribution, where the parameter for the binomial distribution is estimated using a generalized linear model with a logit link (i.e., without a random effect for communities). In this sensitivity analysis, the imputed outcomes are identical for both community structures, whereas the imputation models in the main analysis include a random effect for the community, allowing for slightly different imputed outcomes between the CFG and LE methods. Results and discussion of this sensitivity analysis can be found in the Appendix B.2. In a third sensitivity analysis, we remove communities that have IPWs that are extreme (i.e., very close to zero or very large). Results and discussion can be found in the Appendix C.

## 3. Results

Baseline characteristics of the study population, including demographics, substance use, study recruitment type, and other health-related characteristics, are shown in Table 1. All participants were adults between the ages of 18 and 71. Most participants were male (78%), had a high school education or more (69%), were unemployed or unable to work (70%), were not married (85%), or were of Greek nationality (88%). Seventy-four (27%) participants reported homelessness. In the 6 months prior to the baseline visit, 90% reported having injected drugs. Daily use of cocaine and daily use of alcohol were reported among 20% and 5% of participants, respectively. Of the participants, 14% reported the use of crack, and 22% reported the use of methamphetamines. The majority of participants (70%) reported heroin use. Fifty-one percent of participants reported being unable to get medical care when they needed it, and fifty-two percent had a positive HIV status as ascertained by TRIP. Seventy-five percent of participants reported HIV injection risk behaviors in the 6 months prior to baseline.

**Figure 1.**
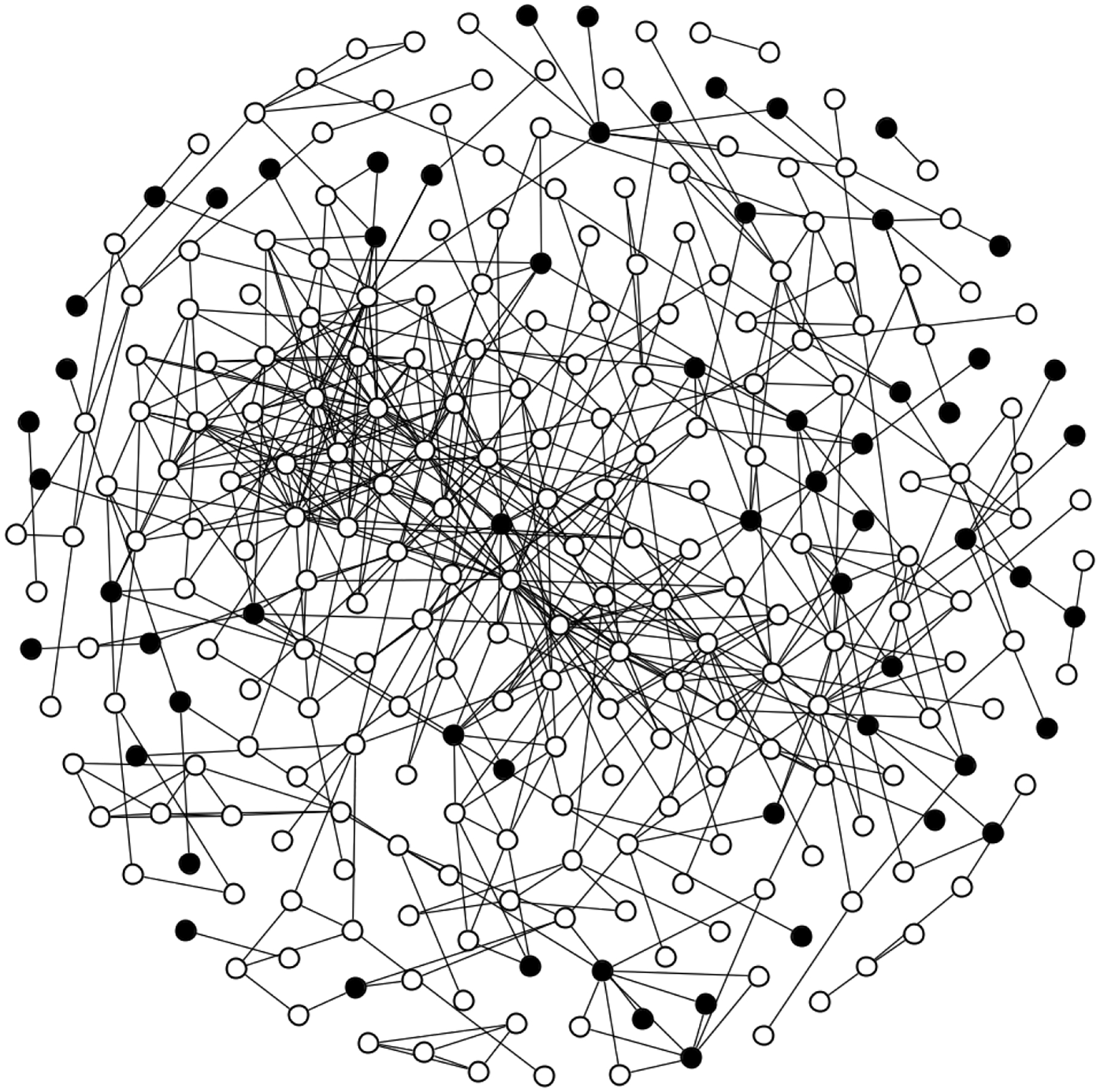
Visualization of the Transmission Reduction Intervention Project network illustrated as a graph. Shaded nodes represent 61 participants treated with MOUD within 6 months prior to the baseline interview, and unshaded nodes represent 216 participants who were not treated with MOUD within 6 months prior to the baseline interview.

The observed TRIP network includes 356 participants with 829 dyadic connections. After removing duplicate edges and 79 (22%) participants who did not share edges with anyone else in the study, the network includes 277 nodes and 542 dyadic connections. Among the 277 participants, there are 7 (2.5%) participants with missing data imputed for covariates and 56 (20%) participants with missing data imputed for the outcome. One participant enrolled in the TRIP study twice and, therefore, had two baseline records and two follow-up records. We include the baseline and follow-up records that are closest to being 6 months apart (the follow-up period for the TRIP study). All edge information from both of these baseline records for this participant is included in the analysis. Figure 1 illustrates the network using unshaded nodes to represent participants who were not treated with MOUD at baseline and shaded nodes to represent participants who were treated with MOUD at baseline. Black lines connecting the nodes represent participants who were drug use or sexual partners, i.e., if they injected drugs together or had sex; injected or had sex in each other’s presence; or injected, used drugs or had sex with the same people; or were reported to have frequented the same venues to use drugs, have sex, or meet new sex partners. Participants sharing edges indicate the possibility of spillover, where participants who received MOUD treatment (i.e., the shaded nodes) may affect the HIV injection risk behaviors of other untreated participants (i.e., the unshaded nodes). As illustrated in Figure 1, the TRIP network is highly connected with many edges connecting treated and untreated participants.

**Figure 2.**
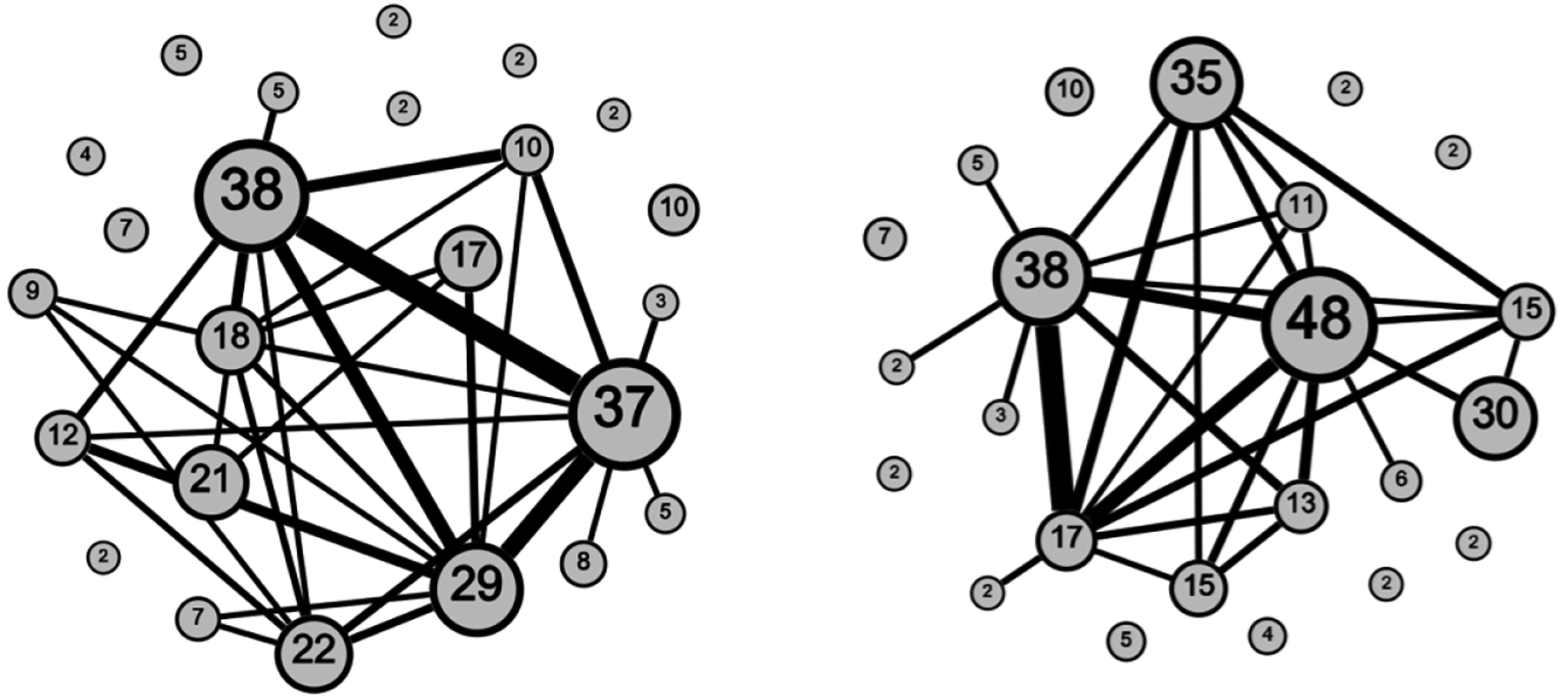
Community structure in the TRIP network found using the CFG method (**left**) and the LE method (**right**). Nodes in this figure represent the communities (24 under CFG method, 23 under LE method), and edges represent connections between participants across those communities. The node labels indicate the number of participants in the community. Data were collected in Athens, Greece, from 2013 to 2015.

We used community detection to determine non-overlapping groupings of nodes (i.e., subnetworks) in the network. Figure 2 illustrates the identified community structure for both the CFG and LE methods. The labels indicate the number of nodes (participants) in the community, and the width of the edges is scaled to express the number of edges (i.e., connections) between communities. As illustrated in Figure 2, under both the CFG (left) and LE (right) methods, there are eight communities detected that had no participants sharing edges with participants in other communities. The remaining communities had varying levels of connectivity between them, with greater connectivity between the largest communities using either community detection method. The CFG method resulted in 24 communities and a community structure with 451 edges within communities and 91 edges between them. The LE method also resulted in 24 communities. One of the communities from the LE method had only one participant, and therefore spillover was not possible in this community. As a result, this participant’s records were not included in any of the models with communities identified from the LE method. The LE method resulted in a community structure with 424 edges within communities and 117 edges between them. The Tchetgen Tchetgen and Coull diagnostic test showed that the normal distribution was a reasonable fit for the random effects (p=0.99 for communities identified by the CFG method, p=0.99 for the LE method) [46].

**Figure 3.**
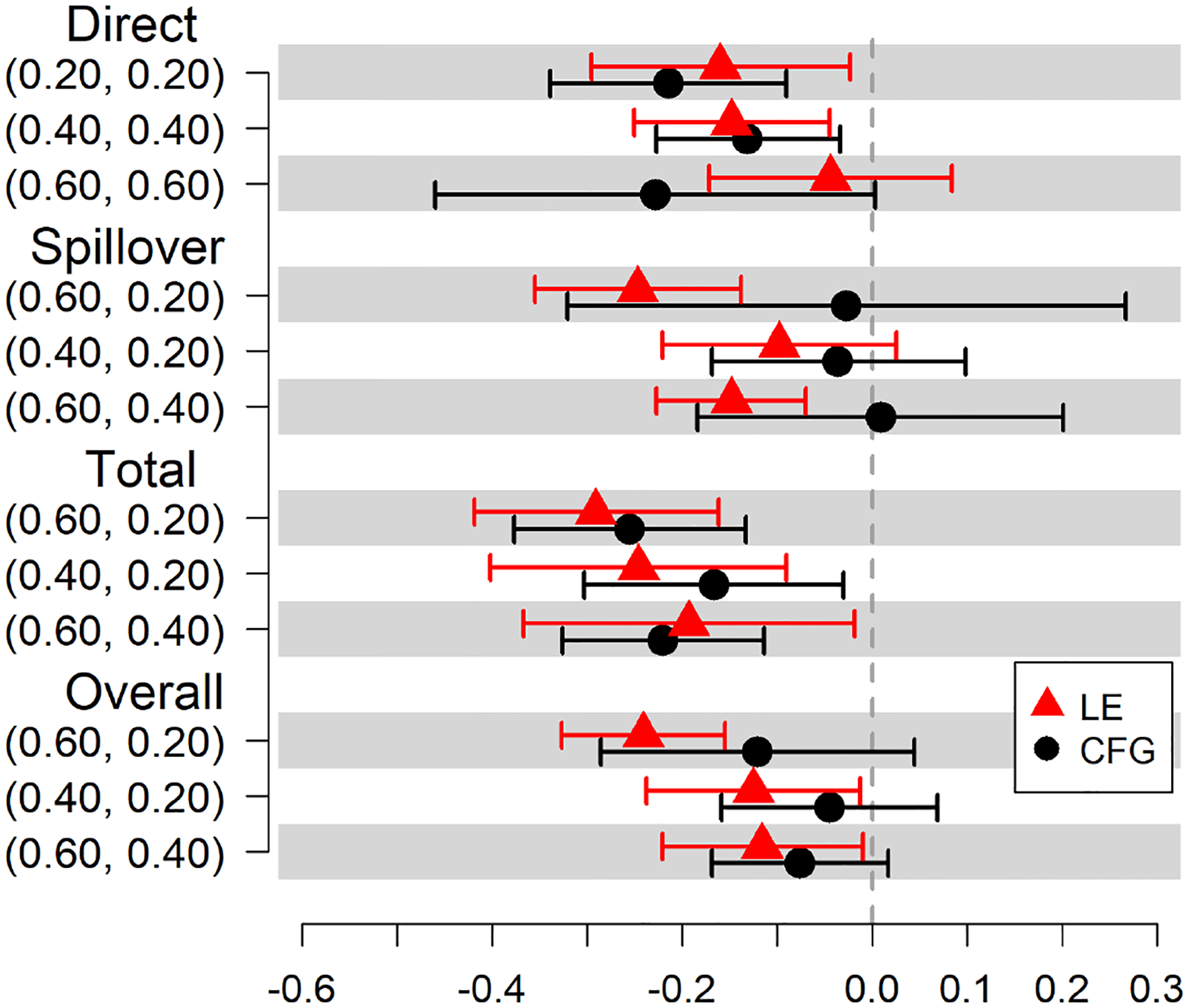
Estimated risk differences with 95% CIs of the estimated effects of MOUD on the likelihood of engaging in HIV risk behavior in TRIP.

We estimate the direct, spillover, total, and overall effects of MOUD treatment on self-reported HIV injection risk behavior at 6 months after baseline. The estimated causal effects on the risk difference scale under both community detection methods are displayed in Table 2, and shown graphically in Figure 3. When communities are identified using the CFG method, we expect 21 fewer reports of HIV injection risk behaviors per 100 persons (95% Confidence Interval (CI) = −0.34, −0.09) at the 6-month visit among treated participants compared to untreated participants with 20% of participants treated in the community. When communities are identified using the LE method, the estimated direct risk reduction is attenuated to 16 per 100 persons (95% CI = −0.30, −0.02). When communities are identified using the CFG method, we expect 3 fewer reports of HIV injection risk behaviors per 100 persons (95% CI = −0.32, 0.27) at the 6-month visit in untreated participants if community coverage is 60% compared to 20%. When communities are identified using the LE method, the estimated spillover risk reduction is 25 per 100 persons (95% CI = −0.35, −0.14). When communities are identified using the CFG method, we expect 26 fewer reports per 100 persons (95% CI = −0.38, −0.13) at the 6-month visit under treatment in communities with 60% coverage compared to under no treatment in communities with 20% coverage. The estimated total risk reduction is 29 per 100 persons (95% CI = −0.42, −0.16) when communities are identified using the LE method. For the estimated overall effect, 12 fewer reports per 100 persons at the 6-month visit would be expected if 60% of participants in the community are treated compared to if only 20% are treated when communities are identified using the CFG method (95% CI = −0.29, 0.04). The estimated overall risk reduction is 24 per 100 persons (95% CI = −0.33, −0.16) when communities are identified using the LE method.

There are differences in the magnitudes of estimated risk differences based on the community detection method used; however, few changes in direction of the effects are observed. Causal estimates under the LE method are significant more times than under the CFG method. The sensitivity analysis shows that the CFG method and the LE method result in estimated risk differences that differ at most by 22% (see Table 2: Spillover (0.60, 0.20)).

**Figure 4.**
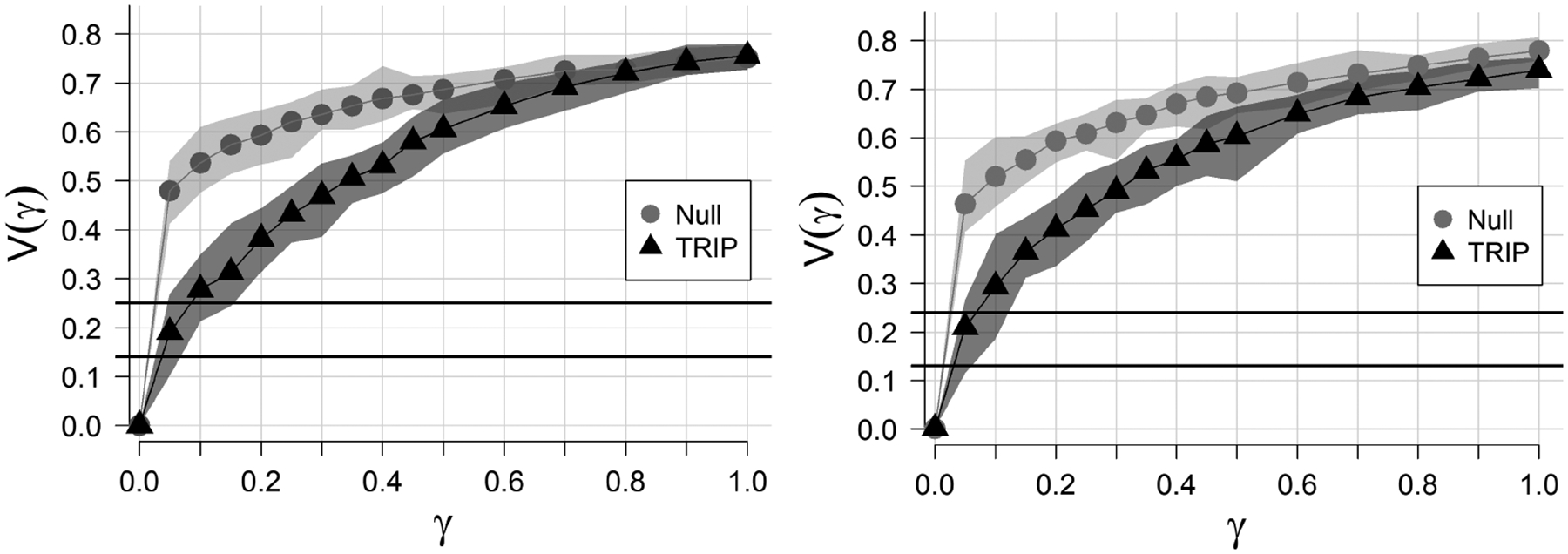
Perturbation plots for the Transmission Reduction Intervention Project network. Robustness of community structure to changes in the proportion of randomly reassigning edges. Communities were detected using the CFG method (**left**) and the LE method (**right**). The light gray area represents the VOI (variation of information) band for the null network, and dark gray represents the VOI band for the TRIP network. Horizontal lines corresponding to the value of V(γ) if 10% and 20% (lower and upper lines, respectively) of nodes were randomly reassigned to other communities. Data were collected in Athens, Greece, from 2013 to 2015.

The V(γ) curves (or VOI curves) in Figure 4 illustrate the significance of the network community structure in TRIP, i.e., the robustness of the community structure to random edge reassignment. Each point represents the average VOI, or $\overset{‾}{V}(\gamma )$, of 30 perturbed graphs. The upper and lower bounds of the bands around the points represent $V_{\text{max}}(\gamma )$ and $V_{\text{min}}(\gamma )$, of the 30 perturbed graphs, respectively. We include the maximum and minimum to illustrate the variation of V(γ). A VOI band for a null network, which is defined to have no community structure, is included as a reference for comparison against the TRIP network. The light gray VOI band represents the null network, and the dark gray VOI band represents the TRIP network. Also included are horizontal lines corresponding to the value of VOI if 10% and 20% (lower and upper lines, respectively) of nodes were randomly reassigned to other communities.

For both the CFG and the LE methods, we observe a possibly meaningful departure of the TRIP network from the null network, indicating a possible presence of community structure in the TRIP network. However, if we consider the value of γ at which $\overset{‾}{V}(\gamma )$ for TRIP crosses the horizontal lines, the community structure is still highly sensitive to small perturbations. The value of γ at which the VOI curve crosses the lower line (or upper line) represents the proportion of edges that would have to be reassigned in the network for 10% (or 20%) of the nodes to be assigned to different communities, respectively. For both community detection methods, less than 5% (or less than 10%) of the edges must be perturbed before 10% (or 20%) of nodes are reassigned to different communities, respectively. This indicates a potentially non-significant community structure and a possible explanation for the sensitivity of point estimates of the direct, spillover, total, and overall effects to the choice of the community detection method.

**Figure A1.**
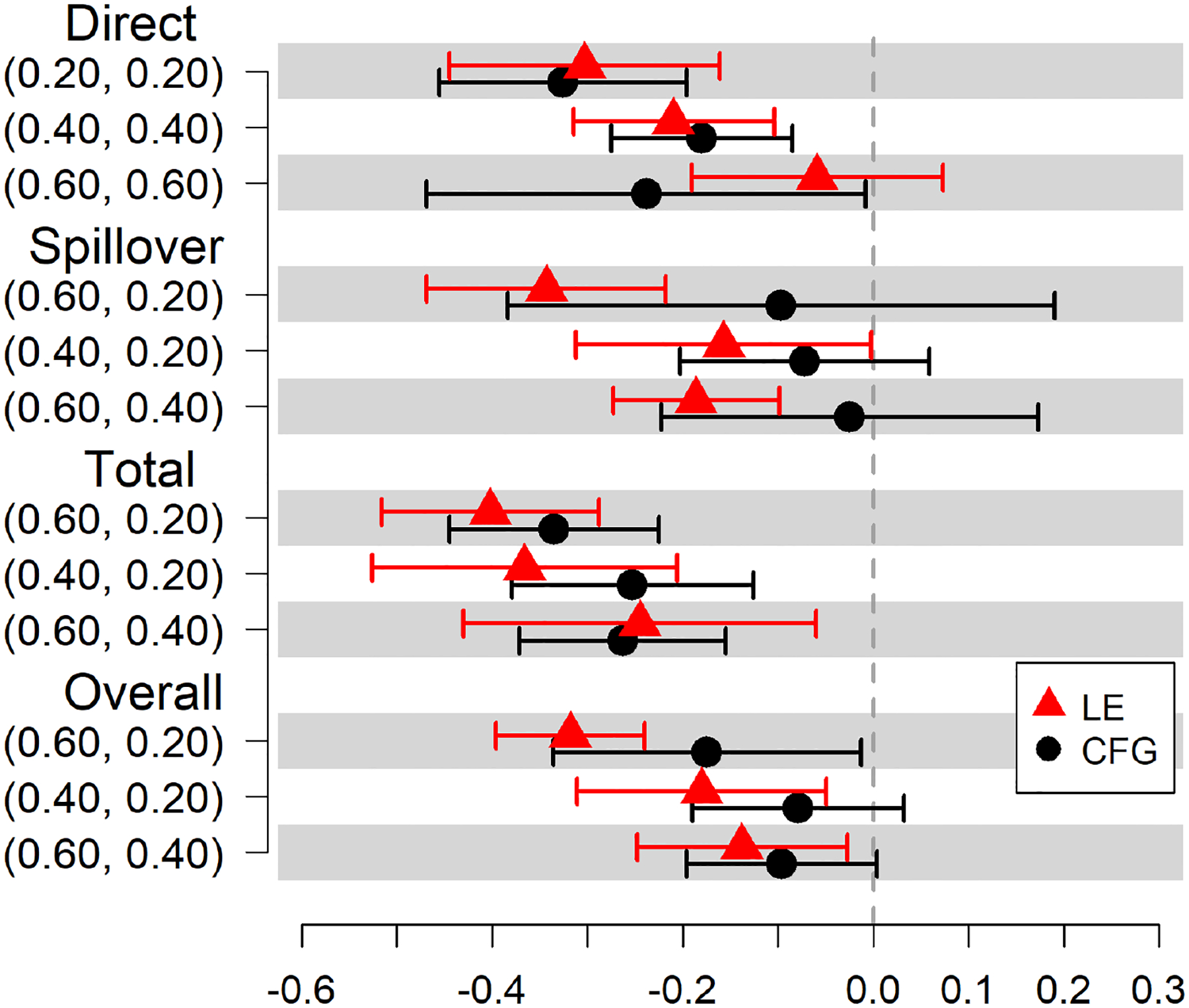
Estimated risk differences under a best-case scenario with 95% CIs of the estimated effects of MOUD (received vs. not received) on the likelihood of engaging in HIV risk behavior in TRIP.

**Figure A2.**
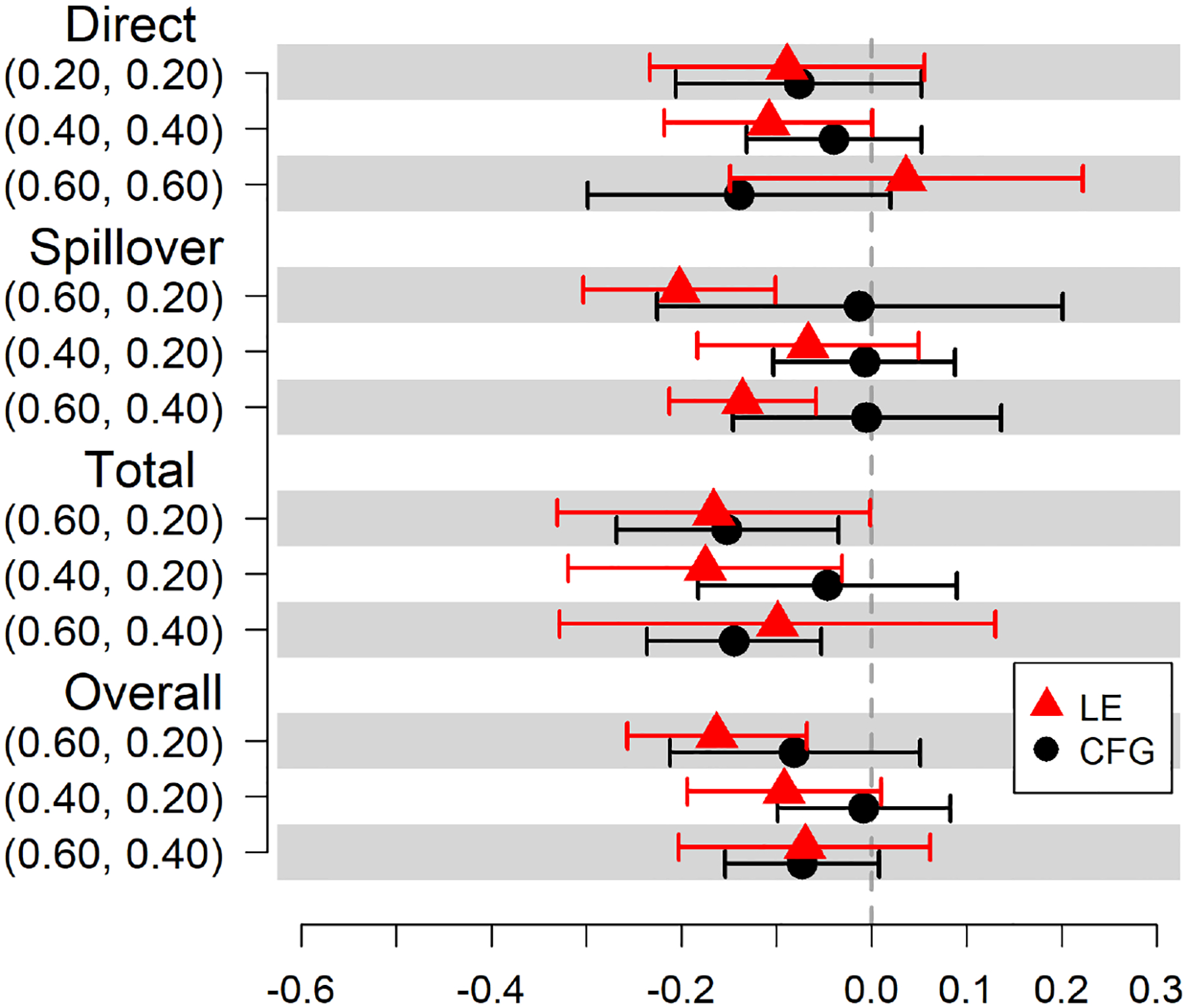
Estimated risk differences under a worst-case scenario with 95% CIs of the estimated effects of MOUD on the likelihood of engaging in HIV risk behavior in TRIP.

A sensitivity analysis using a best/worst-case scenario approach demonstrated that the estimated effects were, at times, sensitive to the missing outcome imputation. However, the estimated causal effects showed a benefit of MOUD for reducing HIV injection risk behaviors. Further details are presented in the Appendix B.1. Under the best-case scenario, we expect 34 fewer reports of HIV injection risk behaviors per 100 persons (95% CI = −0.47, −0.22) at the 6-month visit in untreated participants if community coverage is 60% compared to 20% when communities are identified using the LE method. This estimate is attenuated to 20 fewer reports of HIV injection risk behaviors per 100 persons (95% CI = −0.30, −0.10) at the 6-month visit under the worst-case scenario (shown in Tables A2 and A3, and graphically in Figures A1 and A2). As expected, the best-case scenario shows a greater reduction in HIV injection risk behaviors, and the worst-case scenario shows a smaller reduction than in the main analysis, where we estimate 24 fewer reports of HIV injection risk behaviors if community coverage is 60% compared to 20% under the LE method.

An additional sensitivity analysis shows that when the imputed outcomes are identical for both community structures (i.e., no fixed or random effect for the community is included in the missing outcome imputation model), the causal effect estimates are sensitive to the community detection method. Under the CFG method, we expect 9 fewer reports of HIV injection risk behaviors per 100 persons (95% CI = −0.30, 0.12) at the 6-month visit in untreated participants if community coverage is 60% compared to 20%. This estimate is 26 fewer reports of HIV injection risk behaviors per 100 persons (95% CI = −0.37, −0.16) at the 6-month visit under the LE method. Results and discussion of this sensitivity analysis can be found in the Appendix B.2.

**Figure A4.**
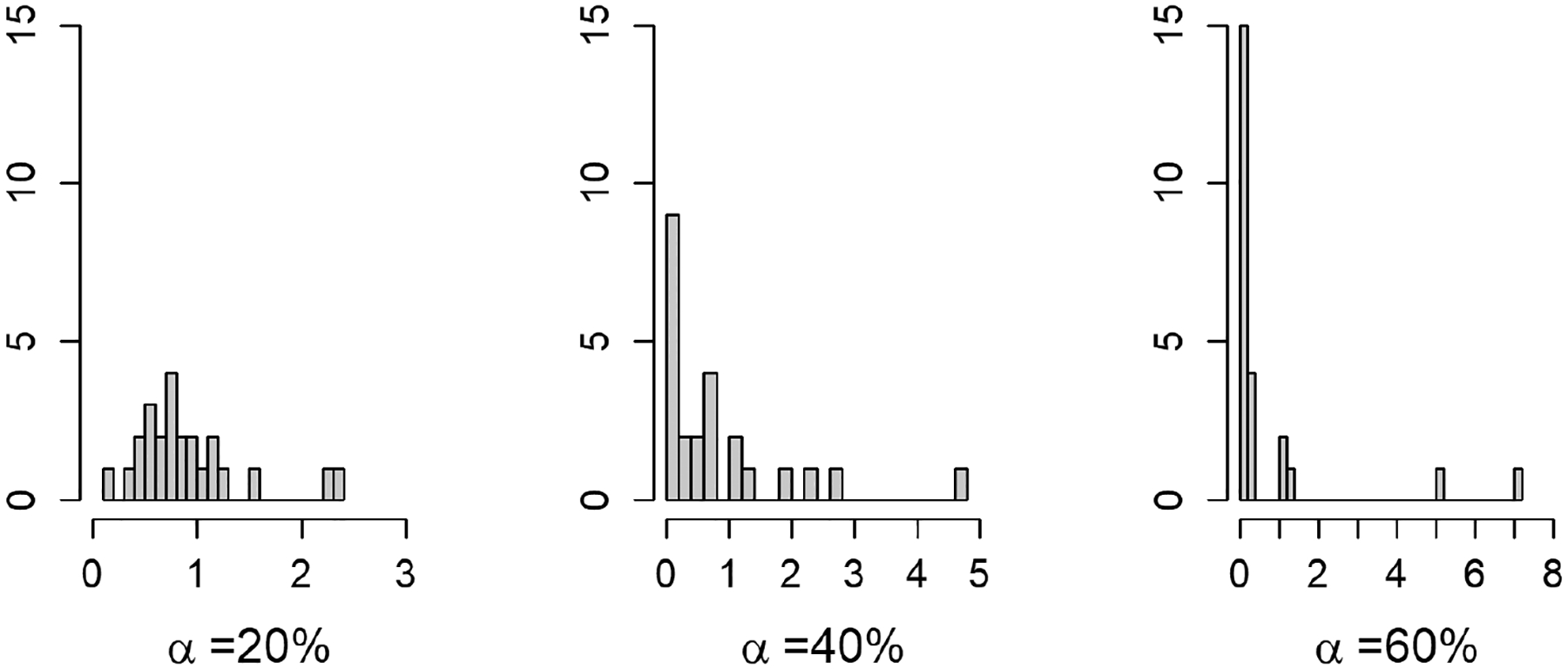
Histograms showing the distribution of the community-level inverse probability weights at each level of treatment coverage. Communities are detected using the CFG method.

**Figure A5.**
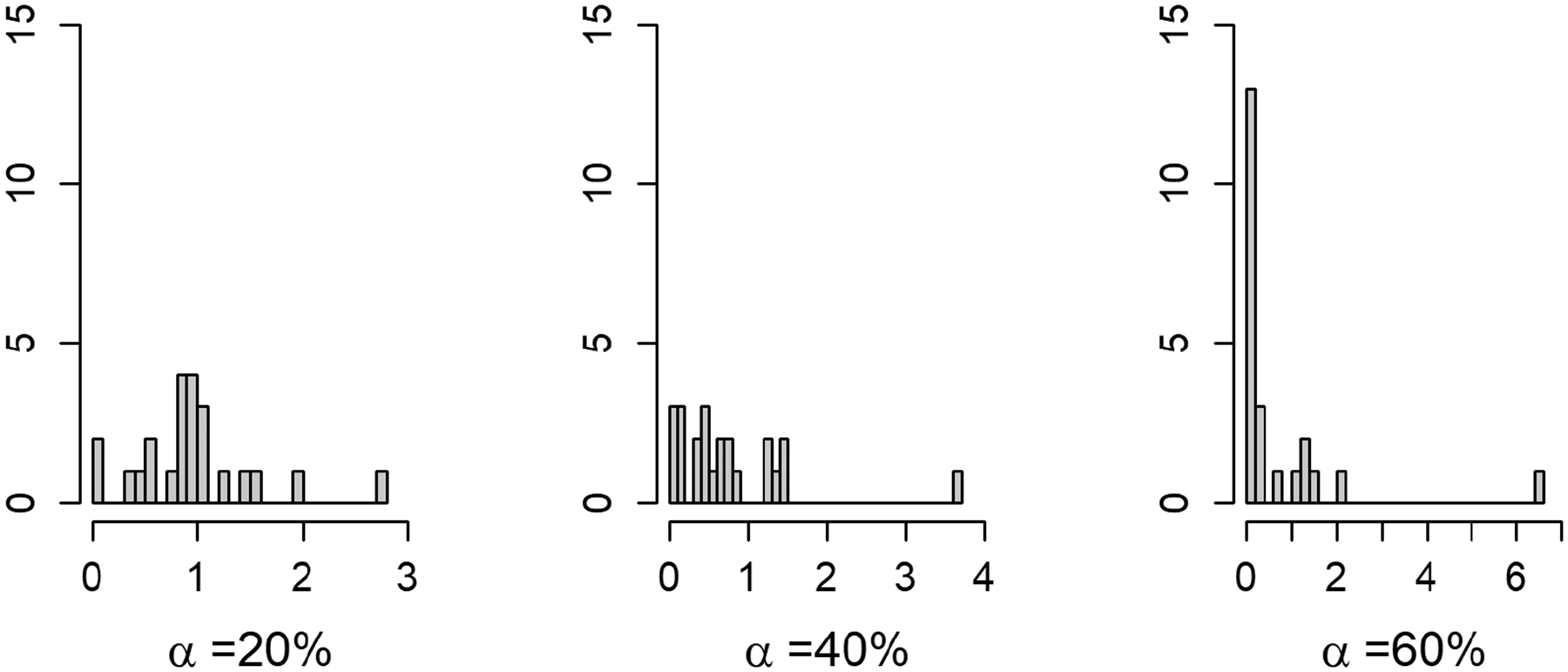
Histograms showing the distribution of the community-level inverse probability weights at each level of treatment coverage. Communities are deteacted using the LE method.

**Figure A6.**
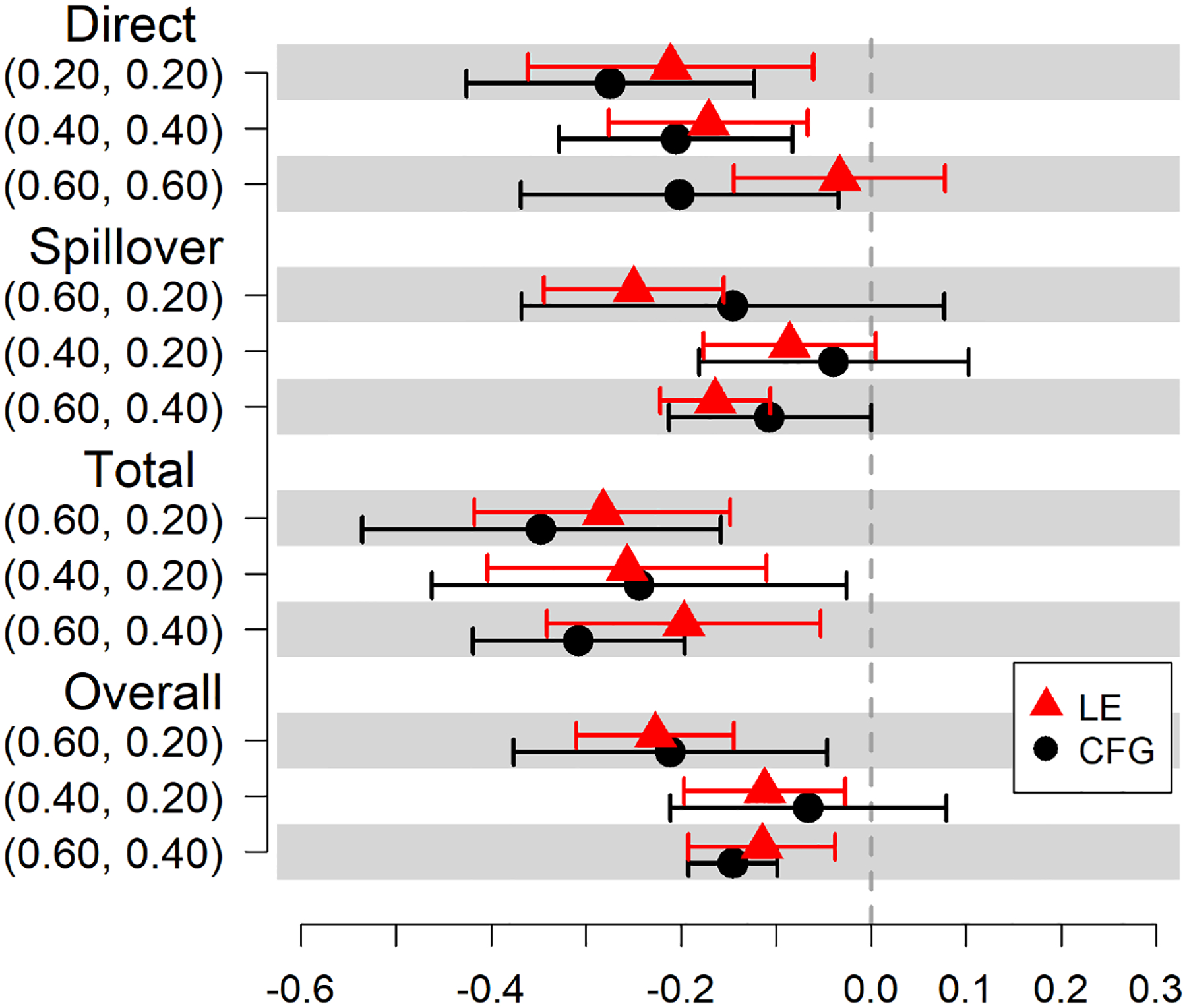
Estimated risk differences with 95% CIs of the estimated effects of MOUD on the likelihood of engaging in HIV risk behavior in TRIP. Communities with IPWs close to zero are removed.

In a third sensitivity analysis, we estimate the same causal effects as in the main analysis but with communities removed which have extreme IPWs. Four of the community-level IPWs are close to zero (i.e., <0.001) under the CFG method and three are close to zero under the LE method. None of the IPWs are especially large (all <8, see Figures A4 and A5). Under the CFG method, after removing the four communities with IPWs close to zero, we expect 15 fewer reports of HIV injection risk behaviors per 100 persons (95% CI = −0.37, 0.08) at the 6-month visit in untreated participants if community coverage is 60% compared to 20%. This estimate is 25 fewer reports of HIV injection risk behaviors per 100 persons (95% CI = −0.34, −0.16) at the 6-month visit after removing the three communities with IPWs close to zero under the LE method (see Table A5 and Figure A6). The estimated reduction in reports of HIV injection risk behaviors is greater than in the main analysis under the CFG method (where we estimate 3 fewer reports of HIV injection risk behaviors per 100 persons) but similar to the main analysis under the LE method. Further details are presented in the Appendix C.

## 4. Discussion

By employing causal inference methods for observational studies with spillover in a network setting, we estimate both direct and spillover effects of MOUD on HIV injection risk behaviors among PWID. The estimated causal effects of MOUD treatment using either community detection method are protective for participants who received or were prescribed MOUD, which is consistent with the current literature. Previous studies have shown that individuals treated for OUD are less likely to engage in injection risk behavior [8–13]. We also find that the receipt or prescription of MOUD may reduce the risk of self-reported HIV injection risk behavior at the 6-month follow-up visit for the drug use and sexual contacts of treated individuals. Notably, in this work, we perform multiple sensitivity analyses to the community structure, and the results are fairly robust. However, the magnitudes of the estimated risk differences are sensitive at times to the community detection method used, namely, for the spillover effects, where estimates under the CFG method are smaller in magnitude and have wider confidence intervals. Variation between the two methods is due to the largest connected component, as the communities outside of the largest connected component are the same for both methods. A large number of edges between communities may imply a violation of the partial interference assumption depending on the number, and type of connections between communities. We also find evidence of a possible non-significant community structure (i.e., where the network can be thought of as one large community without truly separate or distinct groupings within it) in the TRIP network, potentially explaining differences in the magnitudes of causal effect estimates between the two community detection methods.

This paper makes two important contributions to the analysis of PWID networks. First, an analysis of community structure should precede and guide any causal analysis in the presence of spillover in a network study. Based on such an analysis, a decision should then be made as to the most valid method for defining interference sets. If a network has a significant community structure, then community detection may be a valid choice. If a network has a non-significant community structure, then a sensitivity analysis to the community detection methods should be employed, or an alternative approach could be considered. Second, although the magnitudes of the estimated causal effects are sensitive to the community detection method, the results consistently support that MOUD has a protective effect against HIV injection risk behavior for treated individuals and possibly for their contacts in the network. This implies that the protective effect of increasing the prescribing and availability of MOUD may extend beyond the treated individual to other individuals in the PWID network, and the delivery of MOUD may have a further impact by considering the social and risk networks.

A potential limitation of this analysis is bias due to social desirability [56–59]. Further research on social desirability in network settings may lead to insights into how social norms diffuse and sustain through networks, allowing researchers to better account for social desirability when outcomes are self-reported and spillover may be present. An additional limitation is the absence of data on adherence to MOUD, limiting our analysis to a treatment defined by receipt or prescription. One possible spillover mechanism occurs when treated participants do not adhere to their MOUD regimen and instead share some portion with their network contacts (known as “diversion”) [21–23]. Due to lack of data on adherence, we are unable to disentangle the specific spillover mechanisms in this analysis (e.g., diversion, spread of knowledge about injection risk reduction, and behavior modification).

An additional limitation of this analysis is the limited theoretical basis for using community detection to define the interference sets [25,26,29–34]. Although spillover was assessed in settings where interference sets were not known a priori, methods for defining interference sets in this setting are not well established, and further research is needed to develop a theoretical basis to support this approach, including an evaluation of finite-sample performance. More appropriate estimands may be obtained by defining interference sets in a way that better reflects spillover mechanisms, such as using *nearest neighbors* rather than non-overlapping communities that maximize modularity as the interference sets [60,61]. In this approach, spillover only occurs between individuals who share edges (i.e., partnerships), rather than between all individuals in the same community [61]. An additional approach for analyzing the TRIP network could be a chain graph approach that uses *auto-g-computation* for estimation, which allows for arbitrary forms of interference [62]. An area for future research could compare the performance of community detection against other methods such as nearest neighbors and chain graphs in networks with varying characteristics (e.g., significance of community structure, community sizes, and number of communities). Future research may also consider community detection methods that also account for the characteristics of both nodes and edges [63–65].

## Figures and Tables

**Table 1.** Characteristics of 277 participants in Athens, Greece in six months prior to baseline in the Transmission Reduction Intervention Project network with edges defined by sexual and drug use partnerships. Data were collected from 2013 to 2015.

		Treated N (%) 61 (22)	Non-Treated with MOUD N (%) 216 (78)	Overall N (%) 277 (100)
**Demographics**				
Sex^2^	Male	51 (84)	166 (77)	217 (78)
	Female	10 (16)	50 (23)	60 (22)
Homelessness^1,2^	No	48 (79)	155 (72)	203 (73)
	Yes	13 (21)	61 (28)	74 (27)
Highest Level of Education^2^	Primary School or Less	12 (20)	75 (35)	87 (31)
	High School	41 (67)	111 (51)	152 (55)
	At Least Some College	8 (13)	30 (14)	38 (14)
Employment^2^	Unemployed But Looking For Work	10 (16)	54 (25)	64 (23)
	Unable To Work For Health Reasons	36 (59)	93 (43)	129 (47)
	Otherwise	15 (25)	69 (32)	84 (30)
Married^1^	No	54 (89)	182 (84)	236 (85)
	Yes	7 (12)	34 (16)	41 (15)
Nationality	Not Greek	3 (5)	29 (13)	32 (12)
	Greek	58 (95)	187 (87)	245 (88)
Age	Mean (SD)	37 (7)	35 (9)	36 (8)
	Median (Q1, Q3)	37 (32, 41)	34 (30, 39)	34 (30, 40)
	Min, Max	22, 53	18, 71	18, 71
**Substance Use In Last 6 Months**				
Injected Any Drugs^2^	No	7 (11)	20 (9)	27 (10)
	Yes	54 (89)	196 (91)	250 (90)
Daily Use of Cocaine^2^	No	52 (85)	170 (79)	222 (80)
	Yes	9 (15)	46 (21)	55 (20)
Daily Use of Alcohol	No	57 (93)	206 (95)	263 (95)
	Yes	4 (7)	10 (5)	14 (5)
Heroin Use^2^	Never	22 (36)	62 (29)	84 (30)
	A Few Times or More	39 (64)	154 (71)	193 (70)
Crack Use	Never	52 (85)	186 (86)	238 (86)
	A Few Times or More	9 (15)	30 (14)	39 (14)
Methamphetamine Use^2^	Never	40 (66)	176 (82)	216 (78)
	A Few Times or More	21 (34)	40 (19)	61 (61)
**Other Health Related Characteristics**				
Able to Get Medical Care When Needed^1^	Disagree^3^	35 (57)	105 (49)	140 (51)
	Otherwise^4^	26 (43)	111 (51)	137 (49)
HIV Status as Ascertained in TRIP^2^	Negative	27 (44)	107 (50)	134 (48)
	Positive	34 (56)	109 (50)	143 (52)
**Outcome / Recruitment Type**				
Baseline Injection Risk Behavior^1^	No	18 (30)	50 (23)	68 (25)
	Yes	43 (70)	165 (77)	209 (75)
Recruitment Type^2^	Long Term Control	2 (3)	12 (6)	14 (5)
	Marginal Long Term Control	14 (23)	51 (24)	65 (23)
	Network Member of Seed	39 (64)	131 (61)	170 (61)
	Negative Control	1 (2)	6 (3)	7 (3)
	Seed	5 (8)	16 (7)	21 (8)

Column percentages are reported.

1 One participant had missing homelessness status, three participants had missing marital status, one participant had missing status for ability to obtain medical care when needed, and two participants had missing baseline injection risk behavior. Counts and percentages are reported based on imputed values for missing baseline characteristics.

2 Not included in the propensity score models due to model convergence issues related to small cell counts and multicollinearity.

3 “Strongly disagree” or “Somewhat disagree”.

4 “Neither agree nor disagree”, “Somewhat agree”, or “Strongly agree”.

**Table 2.** Estimated risk differences (RDs) with 95% confidence intervals (CIs) of the estimated effects of MOUD (received vs. not received) on the likelihood of engaging in HIV risk behavior in Transmission Reduction Intervention Project, where α and $\alpha^{\prime }$ represent treatment coverage.

	CFG Method		LE Method	
Effect $\left(\alpha ,\alpha^{\prime }\right)$	RD	95% CI	RD	95% CI
Direct (0.20, 0.20)	−0.21	(−0.34, −0.09)	−0.16	(−0.30, −0.02)
Direct (0.40, 0.40)	−0.13	(−0.23, −0.03)	−0.15	(−0.25, −0.04)
Direct (0.60, 0.60)	−0.23	(−0.46, 0.00)	−0.04	(−0.17, 0.08)
Spillover (0.60, 0.20)	−0.03	(−0.32, 0.27)	−0.25	(−0.35, −0.14)
Spillover (0.40, 0.20)	−0.04	(−0.17, 0.10)	−0.10	(−0.22, 0.02)
Spillover (0.60, 0.40)	0.01	(−0.18, 0.20)	−0.15	(−0.23, −0.07)
Total (0.60, 0.20)	−0.26	(−0.38, −0.13)	−0.29	(−0.42, −0.16)
Total (0.40, 0.20)	−0.17	(−0.30, −0.03)	−0.25	(−0.40, −0.09)
Total (0.60, 0.40)	−0.22	(−0.33, −0.11)	−0.19	(−0.37, −0.02)
Overall (0.60, 0.20)	−0.12	(−0.29, 0.04)	−0.24	(−0.33, −0.16)
Overall (0.40, 0.20)	−0.05	(−0.16, 0.07)	−0.13	(−0.24, −0.01)
Overall (0.60, 0.40)	−0.08	(−0.17, 0.02)	−0.12	(−0.22, −0.01)

**Figure A3.**
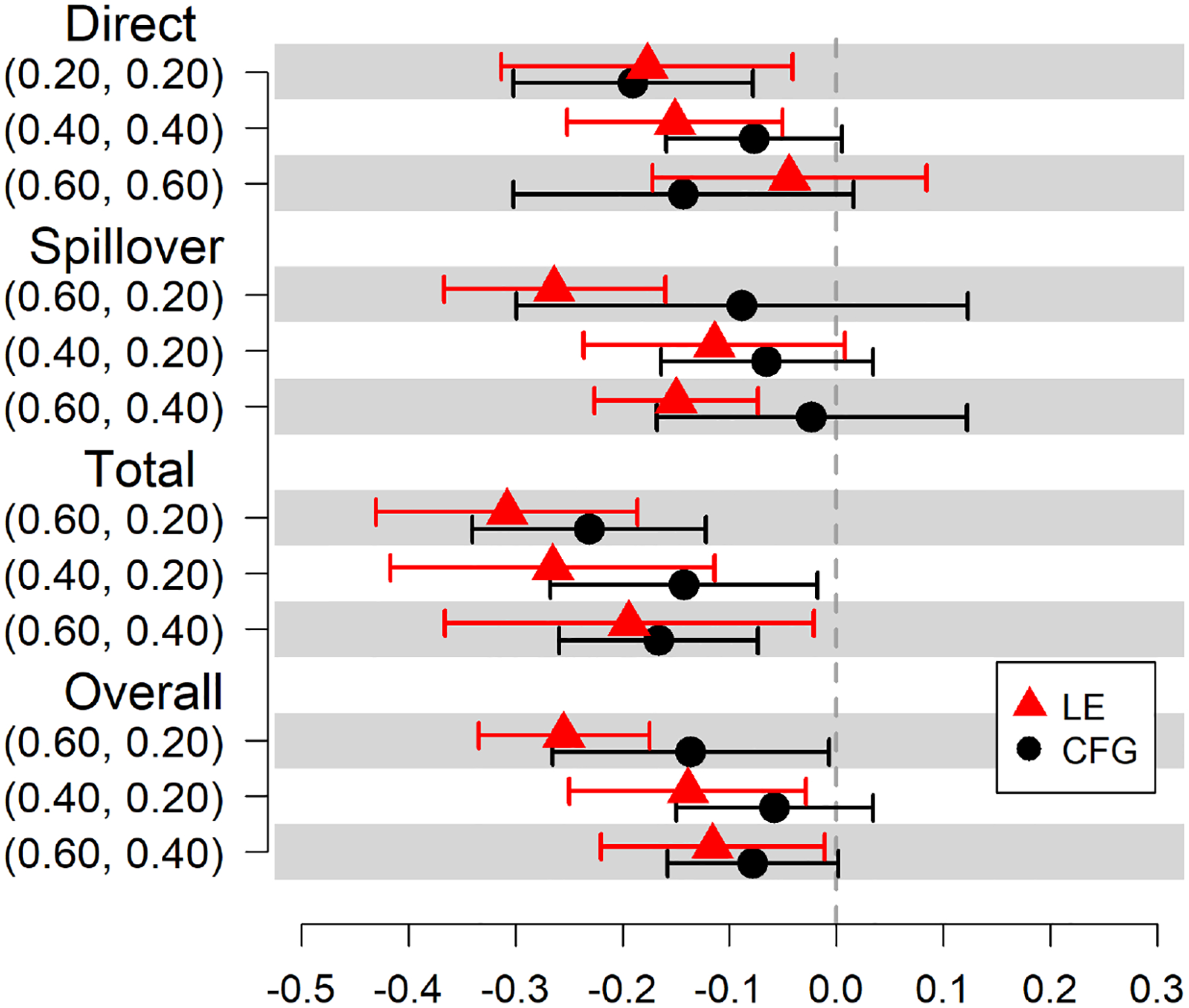
Estimated risk differences with 95% CIs of the estimated effects of MOUD on the likelihood of engaging in HIV risk behavior in TRIP. Missing outcomes are imputed with a random draw from the same binomial distribution for both community detection methods.

## Data Availability

The TRIP datasets are available upon reasonable request to the corresponding author subject to approval by the TRIP investigators. Modeling analysis code is available on GitHub https://github.com/uri-ncipher.

## Appendix A. Variation of Information

Although networks with significant community structure have high modularity, not all networks with high modularity have significant community structure [36]. Thus, the question of the significance of an identified community structure is essentially a question about the robustness of that structure against random reassignment of edges (or perturbations of edges) [36]. Quantifying the robustness of a community structure utilizes concepts from information theory such as *conditional entropy* and *variation of information*. Let $u_{i}$ be the label of the community to which vertex i belongs in partition C, and let $z_{i}$ be the label of the community to which vertex i belongs in partition $C^{\prime }$. The variation of information between partitions C and $C^{\prime }$ is then defined as follows [36]:

(A1)
$$V\left(C,C^{\prime }\right)=H(U|Z)+H(Z|U)$$

(A2)
$$=-\sum_{uz}P(u,z)\text{log}\frac{P(u,z)}{P(z)}-\sum_{uz}P(u,z)\text{log}\frac{P(u,z)}{P(u)}$$

where H(U∣Z) is the conditional entropy of U given Z, i.e., the additional information needed to describe U once Z is known. H(Z∣U) is the additional information needed to describe Z once U is known. The variation of information is the sum of information needed to describe C from $C^{\prime }$ and the information needed to describe $C^{\prime }$ from C. In the main analysis, this quantity is normalized to a 0 to 1 scale by log(n), where n is the number of nodes in the network [36].

## Appendix B. Missing Data

### Appendix B.1. Best/Worst-Case Scenarios

The sensitivity of causal estimates to the missing outcome imputation is assessed in a sensitivity analysis using a best/worst-case scenario approach. This approach provides the largest and smallest possible effect sizes compatible with the observed data [55]. Table A1 shows the joint distribution of MOUD treatment and the baseline outcome (HIV risk behavior within 6 months prior to the baseline interview) among the 56 participants with missing outcome data.

**Table A1.** MOUD treatment and baseline outcome among participants with missing outcome data at follow-up. Percentages are among the 56 participants with missing data for the outcome. The majority of participants with missing outcome data are among the untreated participants who engaged in HIV risk behavior at baseline.

	HIV Risk Behavior at Baseline		
Prescribed MOUD	No	Yes	Total
**Yes**	2 (3.6%)	3 (5.4%)	5 (8.9%)
**No**	7 (12.5%)	44 (78.6%)	51 (91.1%)
**Total**	9 (16.1%)	47 (83.9%)	56 (100%)

A best-case scenario favoring an alternative hypothesis of the protective effect of MOUD treatment against HIV risk behaviors among these 56 participants would imply that untreated participants do experience the outcome, and the treated participants do not experience the outcome. Therefore, for the best-case scenario, we impute the missing outcome with 1 (meaning they did engage in HIV risk behaviors during the 6-month follow-up period) among the 51 participants who were not prescribed MOUD treatment at baseline, and we impute the missing outcome with 0 (meaning they did not engage in HIV risk behaviors during the 6 month follow-up period) among the 5 participants who were prescribed MOUD treatment at baseline. The results under this scenario are reported in Table A2 and shown graphically in Figure A1. A worst-case scenario assuming no effect of MOUD treatment on HIV risk behaviors among these 56 participants would imply that untreated participants do not experience the outcome, and the treated participants do experience the outcome. Therefore, for the worst-case scenario, we impute the missing outcome with 0 (meaning they did not engage in HIV risk behavior during the 6 month follow-up period) among the 51 participants who were not prescribed MOUD treatment at baseline, and we impute the outcome with 1 (meaning they did engage in HIV risk behavior during the 6 month follow-up period) among the 5 participants who were prescribed MOUD treatment at baseline. The results under this scenario are reported in Table A3 and shown graphically in Figure A2. Overall, the results mostly show the protective estimated effects of MOUD treatment on HIV risk behaviors. Similar to the main results reported in this paper, the magnitudes of these effects are sensitive to the community detection method and the estimated spillover effects are greater in magnitude under the LE method.

**Table A2.** Estimated risk differences under a best-case scenario with 95% CIs of the estimated effects of MOUD (received vs. not received) on the likelihood of engaging in HIV risk behavior in TRIP, where α and $\alpha^{\prime }$ represent treatment coverage. Missing outcome data are imputed favoring the alternative hypothesis of a protective effect of MOUD treatment against the likelihood of engaging in HIV risk behavior.

	CFG Method		LE Method	
Effect $\left(\alpha ,\alpha^{\prime }\right)$	RD	95% CI	RD	95% CI
Direct (0.20, 0.20)	−0.33	(−0.46, −0.20)	−0.30	(−0.44, −0.16)
Direct (0.40, 0.40)	−0.18	(−0.28, −0.09)	−0.21	(−0.31, −0.10)
Direct (0.60, 0.60)	−0.24	(−0.47, −0.01)	−0.06	(−0.19, 0.07)
Spillover (0.60, 0.20)	−0.10	(−0.38, 0.19)	−0.34	(−0.47, −0.22)
Spillover (0.40, 0.20)	−0.07	(−0.20, 0.06)	−0.16	(−0.31, 0.00)
Spillover (0.60, 0.40)	−0.02	(−0.22, 0.17)	−0.19	(−0.27, −0.10)
Total (0.60, 0.20)	−0.34	(−0.45, −0.23)	−0.40	(−0.52, −0.29)
Total (0.40, 0.20)	−0.25	(−0.38, −0.13)	−0.37	(−0.53, −0.21)
Total (0.60, 0.40)	−0.26	(−0.37, −0.15)	−0.25	(−0.43, −0.06)
Overall (0.60, 0.20)	−0.17	(−0.34, −0.01)	−0.32	(−0.40, −0.24)
Overall (0.40, 0.20)	−0.08	(−0.19, 0.03)	−0.18	(−0.31, −0.05)
Overall (0.60, 0.40)	−0.10	(−0.20, 0.00)	−0.14	(−0.25, −0.03)

**Table A3.** Estimated risk differences under a worst-case scenario with 95% CIs of the estimated effects of MOUD (received vs. not received) on the likelihood of engaging in HIV risk behavior in TRIP, where α and $\alpha^{\prime }$ represent treatment coverage. Missing outcome data were imputed favoring the null hypothesis of no effect of MOUD treatment on the likelihood of engaging in HIV risk behavior.

	CFG Method		LE Method	
Effect $\left(\alpha ,\alpha^{\prime }\right)$	RD	95% CI	RD	95% CI
Direct (0.20, 0.20)	−0.08	(−0.21, 0.05)	−0.09	(−0.23, 0.06)
Direct (0.40, 0.40)	−0.04	(−0.13, 0.05)	−0.11	(−0.22, 0.00)
Direct (0.60, 0.60)	−0.14	(−0.30, 0.02)	0.04	(−0.15, 0.22)
Spillover (0.60, 0.20)	−0.01	(−0.23, 0.20)	−0.20	(−0.30, −0.10)
Spillover (0.40, 0.20)	−0.01	(−0.10, 0.09)	−0.07	(−0.18, 0.05)
Spillover (0.60, 0.40)	−0.01	(−0.15, 0.14)	−0.14	(−0.21, −0.06)
Total (0.60, 0.20)	−0.15	(−0.27, −0.04)	−0.17	(−0.33, 0.00)
Total (0.40, 0.20)	−0.05	(−0.18, 0.09)	−0.17	(−0.32, −0.03)
Total (0.60, 0.40)	−0.14	(−0.24, −0.05)	−0.10	(−0.33, 0.13)
Overall (0.60, 0.20)	−0.08	(−0.21, 0.05)	−0.16	(−0.26, −0.07)
Overall (0.40, 0.20)	−0.01	(−0.10, 0.08)	−0.09	(−0.19, 0.01)
Overall (0.60, 0.40)	−0.07	(−0.15, 0.01)	−0.07	(−0.20, 0.06)

### Appendix B.2. Aligning Missing Outcome Imputation for CFG and LE

In the main analysis, missing data for the outcome are imputed with a random draw from a conditional binomial distribution, where the parameter for the binomial distribution is estimated using a mixed effects logit model with a random effect for the communities. In this sensitivity analysis, we impute missing data for the outcome with a random draw from a conditional binomial distribution, where the parameter for the binomial distribution is estimated using a logit model with no fixed or random effect for the communities. Similar to the main analysis, this method assumes the missing outcomes are MAR. This approach allows the two parallel analyses (i.e., estimating causal effects under the two community detection methods) to only differ based on the community structures, rather than also having potentially different imputed outcomes. We may consider this analysis to represent a hypothetical scenario in which none of the outcome data are missing, and thus differences between the causal effect estimates are only due to differences in the community structures ascertained by the two community detection methods.

After aligning the outcome imputation for the CFG and LE methods, we expect 19 fewer reports of HIV injection risk behaviors per 100 persons (95% CI = −0.30, −0.08) at the 6-month visit among treated participants compared to untreated participants with 20% of participants treated in the community under the CFG method. When communities are identified using the LE method, the estimated direct risk reduction is 18 per 100 persons (95% CI = −0.31, −0.04). When communities are identified using the CFG method, we expect 9 fewer reports of HIV injection risk behaviors per 100 persons (95% CI = −0.30, 0.12) at the 6-month visit in untreated participants if community coverage is 60% compared to 20%. When communities are identified using the LE method, the estimated spillover risk reduction is 26 per 100 persons (95% CI = −0.37, −0.16). When communities are identified using the CFG method, we expect 23 fewer reports per 100 persons (95% CI = −0.34, −0.12) at the 6-month visit under treatment in communities with 60% coverage compared to under no treatment in communities with 20% coverage. The estimated total risk reduction is 31 per 100 persons (95% CI = −0.43, −0.19) when communities are identified using the LE method. For the estimated overall effect, 14 fewer reports per 100 persons at the 6-month visit are expected if 60% of participants in the community are treated compared to if only 20% are treated when communities are identified using the CFG method (95% CI = −0.26, −0.01). The estimated overall risk reduction is 25 per 100 persons (95% CI = −0.33, −0.18) when communities are identified using the LE method.

The CFG method and LE method result in estimated risk differences that differ at most by 18% (see Table A4 and Figure A3: Spillover (0.60, 0.20)). This sensitivity of causal estimates to the community detection method when estimating spillover highlights the importance of considering the community structure in the network. Similar to the main analysis, the estimates do show a generally protective effect of MOUD on injection risk behaviors for treated individuals and their network contacts, with greater spillover effects when estimated under the LE method.

**Table A4.** Estimated risk differences with 95% CIs of the estimated effects of MOUD (received vs. not received) on the likelihood of engaging in HIV risk behavior in TRIP, where α and $\alpha^{\prime }$ represent treatment coverage. Missing outcomes are imputed with a random draw from the same binomial distribution for both community detection methods.

	CFG Method		LE Method	
Effect $\left(\alpha ,\alpha^{\prime }\right)$	RD	95% CI	RD	95% CI
Direct (0.20, 0.20)	−0.19	(−0.30, −0.08)	−0.18	(−0.31, −0.04)
Direct (0.40, 0.40)	−0.08	(−0.16, 0.00)	−0.15	(−0.25, −0.05)
Direct (0.60, 0.60)	−0.14	(−0.30, 0.02)	−0.04	(−0.17, 0.08)
Spillover (0.60, 0.20)	−0.09	(−0.30, 0.12)	−0.26	(−0.37, −0.16)
Spillover (0.40, 0.20)	−0.07	(−0.16, 0.03)	−0.11	(−0.24, 0.01)
Spillover (0.60, 0.40)	−0.02	(−0.17, 0.12)	−0.15	(−0.23, −0.07)
Total (0.60, 0.20)	−0.23	(−0.34, −0.12)	−0.31	(−0.43, −0.19)
Total (0.40, 0.20)	−0.14	(−0.27, −0.02)	−0.27	(−0.42, −0.11)
Total (0.60, 0.40)	−0.17	(−0.26, −0.07)	−0.19	(−0.37, −0.02)
Overall (0.60, 0.20)	−0.14	(−0.26, −0.01)	−0.25	(−0.33, −0.18)
Overall (0.40, 0.20)	−0.06	(−0.15, 0.03)	−0.14	(−0.25, −0.03)
Overall (0.60, 0.40)	−0.08	(−0.16, 0.00)	−0.12	(−0.22, −0.01)

## Appendix C. Removing Communities with Extreme Estimated Community-Level Propensity Scores

We estimate the same causal effects as in the main analysis after removing communities that have extreme IPWs. Since none of the IPWs are especially large (all <8, see Figures A4 and A5), the removal of communities is only based on IPWs close to zero (<0.001). The communities that are removed tend to be the largest communities identified in the network. Four communities are removed from the CFG analysis, which results in the removal of 125 participants. Three communities are removed from the LE analysis, which results in the removal of 103 participants. Results are shown in Table A5, and graphically in Figure A6. Due to a singular fit for the propensity score model, nationality is removed from the propensity score model under the CFG community detection method.

After removing communities with extreme IPWs, we expect 27 fewer reports of HIV injection risk behaviors per 100 persons (95% CI = −0.43, −0.12) at the 6-month visit among treated participants compared to untreated participants with 20% of participants treated in the community under the CFG method. When communities are identified using the LE method, the estimated direct risk reduction is attenuated to 21 per 100 persons (95% CI = −0.36,−0.06). When communities are identified using the CFG method, we expect 15 fewer reports of HIV injection risk behaviors per 100 persons (95% CI = −0.37, 0.08) at the 6-month visit in untreated participants if community coverage is 60% compared to 20%. When communities are identified using the LE method, the estimated spillover risk reduction is 25 per 100 persons (95% CI = −0.34, −0.16). When communities are identified using the CFG method, we expect 35 fewer reports per 100 persons (95% CI = −0.54, −0.16) at the 6-month visit under treatment in communities with 60% coverage compared to under no treatment in communities with 20% coverage. The estimated total risk reduction is attenuated to 28 per 100 persons (95% CI = −0.42, −0.15) when communities are identified using the LE method. For the estimated overall effect, 21 fewer reports per 100 persons at the 6-month visit are expected if 60% of participants in the community are treated compared to if only 20% are treated when communities are identified using the CFG method (95% CI = −0.38, −0.05). The estimated overall risk reduction is 23 per 100 persons (95% CI = −0.31, −0.14) when communities are identified using the LE method. For the CFG method, causal effect estimates tend to be greater in magnitude than in the main analysis, although spillover effects are similarly non-significant. Causal effect estimates under the LE method are similar to the main analysis, showing a protective effect of MOUD against HIV injection risk behavior for those PWID treated with MOUD as well as their risk contacts in the network.

**Table A5.** Estimated risk differences with 95% CIs of the estimated effects of MOUD (received vs. not received) on the likelihood of engaging in HIV risk behavior in TRIP, where α and $\alpha^{\prime }$ represent treatment coverage. Communities with IPWs close to zero are removed.

	CFG Method		LE Method	
Effect $\left(\alpha ,\alpha^{\prime }\right)$	RD	95% CI	RD	95% CI
Direct (0.20, 0.20)	−0.27	(−0.43, −0.12)	−0.21	(−0.36, −0.06)
Direct (0.40, 0.40)	−0.21	(−0.33, −0.08)	−0.17	(−0.28, −0.07)
Direct (0.60, 0.60)	−0.20	(−0.37, −0.03)	−0.03	(−0.14, 0.08)
Spillover (0.60, 0.20)	−0.15	(−0.37, 0.08)	−0.25	(−0.34, −0.16)
Spillover (0.40, 0.20)	−0.04	(−0.18, 0.10)	−0.09	(−0.18, 0.00)
Spillover (0.60, 0.40)	−0.11	(−0.21, 0.00)	−0.16	(−0.22, −0.11)
Total (0.60, 0.20)	−0.35	(−0.54, −0.16)	−0.28	(−0.42, −0.15)
Total (0.40, 0.20)	−0.24	(−0.46, −0.03)	−0.26	(−0.40, −0.11)
Total (0.60, 0.40)	−0.31	(−0.42, −0.20)	−0.20	(−0.34, −0.05)
Overall (0.60, 0.20)	−0.21	(−0.38, −0.05)	−0.23	(−0.31, −0.14)
Overall (0.40, 0.20)	−0.07	(−0.21, 0.08)	−0.11	(−0.20, −0.03)
Overall (0.60, 0.40)	−0.15	(−0.19, −0.10)	−0.12	(−0.19, −0.04)

## Abbreviations

CFG: Cluster-fast-greedy
CI: Confidence Interval
DE: Direct Effect
HIV: Human Immunodeficiency Virus
LE: Leading-eigenvector
MOUD: Medications for Opioid Use Disorder
OE: Overall Effect
OUD: Opioid Use Disorder
PWID: People Who Inject Drugs
RD: Risk Difference
SE: Spillover Effect
TE: Total Effect
TRIP: Transmission Reduction Intervention Project
VOI: Variation of Information
